# Effect of High-Fat Diets on Oxidative Stress, Cellular Inflammatory Response and Cognitive Function

**DOI:** 10.3390/nu11112579

**Published:** 2019-10-25

**Authors:** Bee Ling Tan, Mohd Esa Norhaizan

**Affiliations:** 1 Department of Nutrition and Dietetics, Faculty of Medicine and Health Sciences, Universiti Putra Malaysia, Serdang 43400, Selangor, Malaysia; tbeeling87@gmail.com; 2 Research Centre of Excellent, Nutrition and Non-Communicable Diseases (NNCD), Faculty of Medicine and Health Sciences, Universiti Putra Malaysia, Serdang 43400, Selangor, Malaysia; 3 Laboratory of Molecular Biomedicine, Institute of Bioscience, Universiti Putra Malaysia, Serdang 43400, Selangor, Malaysia

**Keywords:** cognitive impairment, high-fat diet, inflammation, neurodegeneration, oxidative stress

## Abstract

Cognitive dysfunction is linked to chronic low-grade inflammatory stress that contributes to cell-mediated immunity in creating an oxidative environment. Food is a vitally important energy source; it affects brain function and provides direct energy. Several studies have indicated that high-fat consumption causes overproduction of circulating free fatty acids and systemic inflammation. Immune cells, free fatty acids, and circulating cytokines reach the hypothalamus and initiate local inflammation through processes such as microglial proliferation. Therefore, the role of high-fat diet (HFD) in promoting oxidative stress and neurodegeneration is worthy of further discussion. Of particular interest in this article, we highlight the associations and molecular mechanisms of HFD in the modulation of inflammation and cognitive deficits. Taken together, a better understanding of the role of oxidative stress in cognitive impairment following HFD consumption would provide a useful approach for the prevention of cognitive dysfunction.

## 1. Introduction

The prevalence of obesity worldwide has doubled from 1980 to 2008 [1]. Individuals who are overweight or obese are at risk of developing metabolic syndrome (hyperlipidemia, type 2 diabetes, hypertension, and hypercholesterolemia) [2] and psychiatric conditions, for instance depression [3] and anxiety disorders [4]. Dietary intake of fat has markedly increased from 1991 to 2008 [5]. Substantial evidence highlights the detrimental impact of diets high in saturated fat [6], and extensive research has shown that diet-induced obesity potentially leads to memory impairment in rodents [7]. Mice fed a long-term high-fat diet (HFD) show reduced learning and memory performance [8], as well as depressive- [9,10] and anxiety-like behaviors [11,12].

Oxidative stress plays a crucial role in the development of numerous diseases [13]. Reactive nitrogen species (RNS) and reactive oxygen species (ROS) are continuously produced in the body via mitochondrial bioenergetics and oxidative metabolism [14]. Compelling evidence reveals that overproduction of ROS causes cumulative oxidative damage to macromolecules, including DNA, proteins, and membrane lipids [15], leading to neuronal death and affecting the healthspan of several organ systems [16]. High consumption of dietary fat contributes to obesity, which induces a permanent state of inflammation via the generation of white adipose tissue that secretes proinflammatory factors [17]. A previous study demonstrated that activated immune cells produce high levels of ROS, primarily mediated by nuclear factor kappa B (NF-κB) and proinflammatory cytokines [18].

Oxidative stress plays a prominent role in the development of neurodegenerative diseases [19]. The brain is highly sensitive and vulnerable to oxidation due to the presence of large amounts of unsaturated fatty acids and oxygen that serve as a substrate for lipid peroxidation [20]. Peroxidation products of fatty acids are among the biomarkers of oxidative stress in neurodegenerative diseases, along with protein nitration and carbonylation, and RNA and DNA oxidative damages [21]. Neurodegenerative diseases are often linked to abnormal protein aggregation. Such abnormal protein aggregation is able to induce oxidative stress through ROS production and mitochondria dysfunction [22,23]. Food is a vitally important energy source; it provides direct energy and affects brain function. Therefore, the role of high-fat diet (HFD) modulating neurodegeneration and oxidative stress is worthy of further discussion. This review aims to explore the associations and molecular mechanisms of HFD in modulating cognitive impairment and inflammation.

## 2. HFD-Mediated Oxidative Stress

Diets high in nutrient-dense foods, monounsaturated fatty acids (MUFAs), and dietary fibers have recently been replaced by diets high in saturated fats and refined sugars [24]. Fatty acids interact with various transcriptional factors to trigger downstream signaling pathways [25]. Among all transcription molecules, peroxisome proliferator-activated receptor (PPAR) is a ligand-activated transcription factor that acts as a sensor for lipid-regulating proteins [26]. Polyunsaturated fatty acids (PUFAs) interact with sterol regulatory element binding proteins (SREBP) and transcription factors in the liver may modulate genes associated with their synthesis and facilitate phospholipid, fatty acid, and cholesterol uptake [27]. Studies have revealed a positive association between body adiposity and HFD (r = 0.57, *p* = 0.0002), particularly saturated fat [28]. High body adiposity may induce the production of ROS, accompanied by elevated adipokine and tumor necrosis factor alpha (TNF-α) secretion, which promotes chronic inflammation [29].

Fatty acids are involved in catabolism through peroxisomal β-oxidative and mitochondrial pathways [30]. Mitochondrial β-oxidation is primarily responsible for the degradation of long, medium, and short chain fatty acids (≤18 C), whereas peroxisomal β-oxidation participates in the catabolism of branch chain fatty acids, unsaturated fatty acids, dicarboxylic acids, and very long chain fatty acids (≥20 C) [31,32]. Additionally, peroxisomal β-oxidation is also involved in the synthesis of certain fatty acids, for instance, docosahexaenoic acid (DHA) [33]. Abnormalities in the peroxisomal β-oxidation pathway have been characterized by an accumulation of very long chain fatty acids and progressive neurological dysfunction, including dementia [31]. In particular, PPARα plays a crucial role in regulating fatty acid β-oxidation gene expression for factors such as *catalase*, acyl-CoA oxidase 1 (*ACOX1*), and carnitine-palmitoyl transferase-I (*CPT-I*) [34].

Indeed, the liver is the predominant organ for fatty acid oxidation in the body [35]. A previous study demonstrated that peroxisomal function was inversely correlated with aging [36]. Reduced ACOX1 expression and peroxisomal fatty acid β-oxidation activity have been observed in the liver of old rats [37]. In another study, Sanguino et al. [38] revealed that aging strongly decreases the activity and expression of PPARα in rat liver. These findings imply that fatty acid β-oxidation activity is reduced with age, subsequently resulting in reduced levels of hepatic PPARα [39].

In addition, several studies reported by O’Brien et al. [40] and Okereke et al. [41] have shown an association (*p* for linear trend = 0.008, odds ratio (OR) with 95% confidence interval (CI) = 1.64) between excessive fat consumption and neurological dysfunction. Data indicated that excessive fat consumption results in a net energy overload, which in turn leads to the expansion of adipose tissue. Sustained chronic fat intake may contribute to adipose tissue dysfunction and metabolic inflammation. When circulating free fatty acids increase, they can induce lipotoxicity to peripheral tissues, such as liver and β-cell dysfunction [40]. The high influx of free fatty acids into the liver triggers very low-density lipoprotein (VLDL) triglycerides production and contributes to the development of dyslipidemia. Subsequently, these impairments can lead to metabolic syndromes. The central nervous system is also adversely affected by elevated levels of circulating free fatty acids and the lipotoxic effects of dyslipidemia [40]. Ultimately, these dysfunctions contribute to neurological disorders, such as mild cognitive impairment and Alzheimer’s disease [40].

Furthermore, HFD increases levels of chylomicrons in the intestine. These chylomicrons enter circulation and cause the generation of free fatty acids, which are taken up by the liver. These hepatic free fatty acids may either enter the mitochondria for β-oxidation or be esterified into triglycerides. Triglycerides either accumulate in hepatocytes as small droplets or produce VLDL, which is then converted into low-density lipoprotein (LDL) [42]. Excessive LDL burden in the blood may form oxidized-LDL (Ox-LDL) due to its excessive accumulation or lack of LDL-receptors in hepatocytes, which in turn is engulfed by macrophages and becomes foam cells. Subsequently, foam cells accumulate in the arterial endothelium to form plaques. Ultimately, these plaques lead to cardiovascular and circulatory disorders and increase blood-brain barrier permeability [43].

Additionally, mitochondrial β-oxidation of free fatty acids is linked to the conversion of oxidized cofactors (FAD and NAD^+^) into reduced cofactors, FADH_2_ and NADH and is thereby reoxidized and restored back into FAD and NAD^+^ by the mitochondrial respiratory chain. During reoxidation, NADH and FADH_2_ transfer electrons to the first complexes of the respiratory chain. These electrons then migrate up to cytochrome c oxidase and combine with oxygen and protons to form water. These intermediates may interact with oxygen to produce increasing levels of ROS and superoxide anion radicals [44,45]. Therefore, overconsumption of fat triggers mitochondrial β-oxidation of free fatty acids, subsequently leading to excess electron flow using cytochrome c oxidase, which increases ROS accumulation. Mitochondria are a crucial cellular source of ROS; they oxidize unsaturated lipids of fat deposits to cause lipid peroxidation. HFD-induced ROS may trigger proinflammatory signaling and activate NF-κB transcriptional factor, and thus inducing NF-κB-dependent proinflammatory molecules, such as interferon-γ (IFN-γ), TNF-α, and inducible nitric oxide synthase (iNOS) [46]. In addition to ROS production, overproduction of nitric oxide (NO) via the activation of iNOS also causes accumulation of RNS [47].

## 3. Association of a High-Fat Diet with Cognitive Function

### 3.1. Animal Study

A previous study has demonstrated that rodents fed a diet high in saturated fatty acids (SFAs) show increased brain inflammatory markers [48]. Increased body weight and adiposity can cause neurological perturbations. These data imply that excess adipose tissue is highly metabolically active and susceptible to the release of proinflammatory mediators [49]. Further, data from a previous study also demonstrated that triglyceride administration impairs hippocampal long-term potentiation [50]. Interestingly, the mice treated with HFD for only one day is sufficient to induce a rapid drop in the performance of episodic memory task [51]. This study further revealed that memory deficits are rapidly reversed when switching mice to a low-fat diet from a HFD [51]. In another study, Duffy et al. [52] found that orexin/ataxin-3 (O/A3) mice, a transgenic mouse model of orexin neurodegeneration, fed with HFD accelerate two-way active avoidance (TWAA) hippocampus-dependent memory task and the onset of neuroinflammation. In a study by Woodie and Blythe [53] focusing on hypercaloric diet (high in fat and fructose) and its effects on cognitive health, the rats fed with a HFD exhibited a relatively high fat pad weight compared to the high-fructose group. This study further demonstrated that high-fructose diet promotes insulin dysregulation, development of hyperlipidemia, and impairs cognitive performance. However, no apparent cognitive deficits were observed in rats fed with a HFD [53]. These observations indicate that an individual part of the hypercaloric diet may cause negative impacts on metabolic syndrome and cognitive function, suggesting the combination of high-fructose and high-fat components may further aggravate physiological issues and be detrimental to human health. In support of this, the animal study has shown that hypercaloric diets altered lipid and energy metabolism similar to clinical diabetes, with elevation of fasting glucose and increased cholesterol levels. This study further revealed that hypercaloric diets can impair spatial learning ability and synaptic plasticity [54]. These adverse effects were also found in rats fed with a HFD, suggesting that insulin resistance is a probable mediator to HFD-induced cognitive deficits [55]. Table 1 shows the effects of a HFD on cognitive function in animal models.

### 3.2. Human Study

A recent study stated that Western-style diets, which are high in refined sugars and saturated fats, have a greater likelihood of inducing memory impairment in healthy subjects [56]. Data from human studies investigating the impact of HFD on cognitive function (Table 2) are limited, but evidence from an intervention study indicates an association between HFD consumption and cognitive disorder. Holloway et al. [57] found that HFD administration (nearly 75% of energy) for five days is sufficient to induce depression and impair retrieval speed and attention. Consistent with a study reported by Holloway et al. [57], Edwards et al. [58] demonstrated that HFD consumption for seven days reduced attention and reaction time in sedentary adult males with fewer than 2 h/week physical activity. Similarly, HFD was also found to induce neuroinflammation. A study reported by Mittal and Katare [59] also demonstrated that high triglyceride levels convey poor cognitive performance in type 2 diabetes patients. Moreover, a study by Okereke et al. [41] also found that increased intake of saturated fats in young adults impairs prospective memory, cognitive function, and memory speed and flexibility, leading to neurological diseases, such as dementia and Alzheimer’s disease in mid and later life. Although many studies have demonstrated that HFD increased the risk of cognitive disorders; not all data demonstrated such a link [60]. Several studies reported by Solfrizzi et al. [61], Cherbuin and Anstey [62], and Roberts et al. [63] did not identify an association between high SFA intake and mild cognitive impairment. Variability in study designs may partly explain the inconsistency in findings. For instance, the variation in means of reporting composition and type of dietary fats and its association with cognitive problems shows a barrier in combining the findings of studies. The investigators used different methods for defining fat types (for example, PUFA from spreads or total PUFA) may address different subgroups (for instance, those with diabetes). Furthermore, the small sample size may increase the likelihood of inconsistent findings between the studies. In fact, the small sample size of study makes it difficult to detect significant associations. The nonsignificant findings could be attributed to the lack of statistical power. In addition, by comparing different study groups with diverse populations and its relationship with cognition may also explain inconsistency in findings. Individuals from different human populations have different proportions of genetic. Genetic variation in a population is derived from a wide assortment of alleles and genes. The persistence of certain populations over time via changing environments is highly dependent on their ability to shift or adapt external conditions.

**Figure 1:**
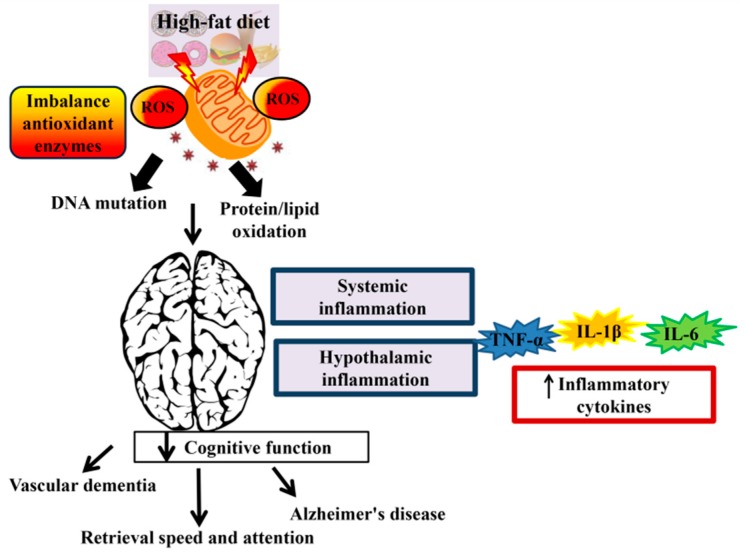
The effect of a high-fat diet (HFD) on cognitive function. Consumption of HFD induces reactive oxygen species (ROS). Accumulation of ROS leads to DNA mutation and protein/lipid oxidation and subsequently reduced the mitochondrial function. Overproduction of reactive species that occur in the mitochondrial DNA can lead to neurodegenerative disease and brain dysfunction. Systemic inflammation and hypothalamic inflammation promotes cognitive decline via secretion of inflammatory cytokines such as tumor necrosis factor alpha (TNF-α), interleukin-1 beta (IL-1β), and interleukin-6 (IL-6).

In addition, data from a Rotterdam cohort study involving 197 cases of dementia, including 29 with vascular dementia, 146 with Alzheimer’s disease, and 22 with other types failed to show adverse outcome of high SFA intake and dementia risk after 6 years of follow-up [72]. Furthermore, data from Washington Heights-Inwood Columbia Aging Project (WHICAP) involving 980 New Yorkers aged 65 years and above, in which 28% of them carried APOE ε4 allele, showed that high SFA intake was not associated with the increased risk of Alzheimer’s disease [73]. Specifically, data demonstrate that compared to an omega-6 PUFA diet, Alzheimer’s disease risk was reduced in subjects consuming an omega-3 PUFA diet [74,75]. In contrast, high omega-3 PUFA intake is not associated with long-term dementia risk [76] (*p* for linear trend = 0.7, hazard ratio (HR) = 0.95; 95% CI = 0.76, 1.19). Taken together, these findings demonstrate an association between HFD and cognitive loss, suggesting potentially causal roles of HFD and brain inflammation in driving cognitive disruption. Figure 1 shows the effects of HFD on cognitive function.

The studies included in Table 1 and Table 2 had to meet the following criteria: exposure to HFD (PUFA, total, saturated, trans fat, or cholesterol) in rodents (rats or mice) or at any adult age in human; endpoints included cognitive function, incident mild cognitive impairment, Alzheimer’s disease, or dementia; or cognitive decline; in studies assessing dietary assessment and determination of cognitive function or outcome (mild cognitive impairment, Alzheimer’s disease, or dementia) or in studies assessing cognitive decline. There was no restriction pertaining to the publication date, sample size or the number of animals, animal strains, ethnicity of participants or on language, race, or gender. Exclusions criteria in animal study should also be considered. First, HFD was not administered. Second, other types of animals (dogs, cats, or sheep) were used. Third, duplicate publications. Moreover, case reports and studies limited to individuals with medical conditions (dyslipidemia or CVD) that are likely to affect intervention trials and cognitive status were excluded because of the confounding or bias in their study designs.

Data from the animal experiments have shown a greater likelihood of inflammation after the administration of HFD (Table 1). Importantly, some research has emerged to suggest the detrimental impact of HFD on gene expression in hypothalamus [66,67,68]. In particular, a HFD led to an upregulation of genes such as toll-like receptor 4 (*TLR4*), *NF-κB*, *Cd68*, *Emr1*, *IL-6*, indoleamine 2,3-dioxygenase (*IDO*), *TNF-α*, and interferon gamma (*IFN-γ*) in rodents, suggesting that these changes were related to diet-induced changes rather than obesity. In line with this, human studies also showed an association between HFD and Alzheimer’s disease, mild cognitive impairment, or dementia (Table 2). These relationships could be partly due to the type or composition of dietary fatty acids that may influence the cognitive function. In fact, the methodological quality of human studies may fail to assess precision outcomes. Most of the human studies using semiquantitative food-frequency questionnaire (FFQ) to measure the habitual intake during the study period and do not account for long-term periods or for day-to-day variation of intake. The measures obtained from the FFQ may not have enough precision to make inferences on the absolute amounts of nutrient intake that linked to the occurrence of cognitive outcomes. Although several limitations and potential publication bias may undermine the validity of the findings, HFD may play a potential role in neurodegenerative disease. Overall, these data imply that HFD consumption, particularly SFA may serve as a stimulus to elevate the inflammatory markers and augmented the inflammatory response, and ultimately lead to brain dysfunction and neurodegenerative disease.

## 4. Mechanisms Responsible for High-Fat Diet-Induced Cognitive Deficits

Substantial evidence suggests that increased oxidative stress and altered apoptosis contribute to the pathogenesis of neurodegenerative diseases [77]. Free radicals are implicated in the progression and development of cognitive deficits by interrupting synaptic transmission, mitochondrial function, neuroinflammation, and axonal transport; all of these factors contribute to neuronal loss in Alzheimer’s and other dementia diseases [78]. In fact, mitochondria are involved in the pathogenesis of numerous neurodegenerative diseases. Mitochondria are the predominant organelles that supply ATP to cells via oxidative phosphorylation, respond to oxidative stress, and synthesize additional key molecules. Mitochondria produce redox enzymes that are required for transferring electrons from one substrate to another, and inefficiencies in this process may lead to ROS production. The central nervous system is highly dependent on mitochondrial function due to its high energy demands. Mutation and ROS production that occurs in the mitochondrial DNA can lead to neurodegenerative disease and brain dysfunction. Indeed, many neurodegenerative diseases have been linked to mitochondrial dysfunction [77].

Accumulation of mitochondria DNA mutations during aging may cause malfunctioning oxidative phosphorylation and an imbalance in antioxidant enzymes. Mitochondrial dysfunction produces the ROS axis and forms a vicious cycle, which is regarded as the basis of the mitochondrial free radical theory of aging. A previous study revealed that ROS plays a critical role in the regulation of several cellular metabolic processes, including antioxidant defense mechanisms and posttranslational modification of proteins. However, oxidative stress in normal homeostasis is disturbed during aging and subsequently increases intracellular ROS levels [79]. Production of oxidative stress affects the mitochondrial defense system and membrane permeability, influences Ca^2+^ homeostasis, deranges the mitochondrial respiratory chain, and causes DNA mutations in mitochondria. This phenomenon triggers neurodegeneration and amplifies neuronal dysfunction [80].

### 4.1. Animal Study

Hippocampal injury is found in HFD-fed animals in response to increased blood-brain barrier permeability [71] due to circulating proinflammatory adipokines and reactive glial cytokine production [81]. Increased blood-brain barrier permeability that allows proinflammatory proteins into the hippocampus can initiate neuroinflammation and stimulate neurodegeneration [82]. A previous study further revealed that feeding mice a HFD increases both the permeability of the blood-brain barrier and markers of hippocampal inflammation, such as microglial activation [65]. The animal study further demonstrated that HFD-induced alterations of hippocampal structure and function [83]. For example, HFD impairs the hippocampus [70], a major region in the brain that plays an important role in memory and learning. Emerging evidence suggests that hypothalamic inflammation represents an initial stage of a vicious feed-forward cycle of central nervous system dysfunction, which in turn causes cognitive decline [84]. In addition to inflammation, alteration of free fatty acid metabolism might contribute to changes in the central nervous system [85].

### 4.2. Human Study

Individuals with obesity or who are overweight are at risk of developing cognitive disorders, metabolic syndrome, and depression. Furthermore, data from a previous study demonstrated that cerebral uptake of free fatty acids is higher in obese individuals compared to healthy individuals [86]. Increased dietary fat not only contributes to hypothalamic injury, but other factors, such as metabolic dysfunction in combination with alteration of triglycerides and free fatty acids, may also affect certain brain regions, particularly the hippocampus [87]. The role of the inflammatory pathway on cognitive impairment will be described in the following section.

## 5. Modulation of Inflammatory Pathways

Inflammation is a critical pathophysiological mechanism underlying dementia and cognitive dysfunction that results in the formation of neurofibrillary tangles and amyloid plaques [88]. Thus, brain function is highly susceptible to inflammatory mediators. Obesity is characterized as an alteration and elevation of the peripheral inflammatory response [89] and peripheral inflammation, which can promote neuroinflammation [90]. Chronic low-grade systemic inflammation has been suggested as a major pathophysiology underlying obesity [91]. Fatty acids stimulate TLR4, lipopolysaccharide (LPS) receptor, and free fatty acids in immune cells, initiating the inflammatory cascade [92].

### 5.1. Animal Studies

Systemic inflammation has been associated with cognitive decline or dementia [93,94]. Systemic inflammation triggers acute neuronal dysfunction via neuronal receptors. Data from an animal study showed that expression of CD200 on neuronal dendrites was decreased with age in response to LPS stimulation [95]. Similarly, aged animals also exhibited a primed microglial response to secondary stimulation of LPS [96]. In another study, deletion of the CX3CL1 receptor (CX3CR1), a neuronal cell surface marker, increased susceptibility to LPS-induced central nervous system inflammation and promoted sickness behavioral responses in aged mice [97]. Villaran et al. [98] explored the impact of systemic inflammation on neuronal cells using LPS and dextran sulfate sodium (DSS). Data indicated that ulcerative colitis induced by DSS aggravates dopaminergic neuronal loss [98].

Additionally, feeding genetic mice HFD may increase IL-1β levels in the hippocampus, which in turn decreases hippocampal cognitive function [69,99,100,101]. The arcuate nucleus (ARC) of the hypothalamus exhibits a lack of effective blood-brain barrier response to circulating factors, such as inflammatory mediators and nutrients [88]. Yet this circulating signal is a crucial driving force in generating central inflammation after HFD administration. For example, TLR4 binds LPS and extracellular lipids to trigger inflammation [102].

Peripheral cytokines can act on the brain to trigger cytokine production [103]. An animal study demonstrated that feeding a diet containing high-fat content induces central inflammation in genetic models of obesity, particularly in the hypothalamus [104]. Data from another animal study further revealed that insensitivity of the leptin receptor [105] upregulated the transcriptional activity of interleukin-6 (IL-6), TNF-α, and IL-1β in the hippocampus compared to control [106]. Another study also reported that feeding 60% of HFD for 20 weeks increased TNF-α expression in the hippocampus [65]. Furthermore, chronic intake of HFD aggravates LPS-induced cytokine expression such as interferon-γ and TNF-α in the hippocampus and IL-6 in the hypothalamus [68]. Taken together, these data suggest that administration of HFD produces more free fatty acids to enter ARC and initiates an inflammatory cascade [107].

### 5.2. Human Studies

Extensive studies have shown that accumulation of white adipose tissue is predominant in sites that produce systemic inflammation [108]. In general, both adipose tissue-resident immune cells and hypertrophied adipocytes contribute to the elevation of circulating levels of proinflammatory cytokines, such as TNF-α, IL-6, IL-1β, and C-reactive protein [109] in obese individuals. Individuals with high waist circumference and waist:hip ratio demonstrated a high IL-6 and C-reactive protein concentrations [110]. In addition, evidence suggests that chronic low-grade inflammation is mediated by T-cells [111]. Data from a cross-sectional study revealed that obese women exhibit high levels of T-cell derived cytokines (IL-17 and IL-23), which are statistically independent of leptin and other inflammatory factors, for instance, macrophage migration inhibitory factor [112]. This observation implies that IL-17 plays a critical role in obesity [112].

Notably, data showed that adipose tissue secretes TNF-α [113]. Cytokines, for instance, IL-6 and IL-1β, were found to disrupt neural circuits involved in memory and cognitive function [114]. Increased plasma IL-12 and IL-6 decline cognitive function and processing speed in the elderly population [99]. Another study conducted by Harrison et al. [115] also demonstrated that induction of systemic inflammation in response to *Salmonella typhi* vaccination in humans reduced spatial memory, suggesting that the medial temporal lobe is involved in modulating systemic inflammation.

HFD promotes cognitive decline via multiple mechanisms, such as increased expression of proinflammatory adipokines (TNF-α and IL-6), upregulated chemotactic adipokines (monocyte chemoattractant protein-1 (MCP-1)), and increased reactive microgliosis and astrocytosis [48]. In this regard, interleukin-1 beta (IL-1β) may modulate neurophysiological mechanisms involved in memory and cognition [116]. Moreover, the detrimental impact of triglycerides was also observed in the brain when pancreatic lipase breaks down triglycerides released from adipocytes into free fatty acids. Additionally, dietary intake of SFAs, such as lauric acid and palmitic acid, was found to trigger inflammatory responses in cultured macrophages [117], as well as microglial and astrocytic signaling pathways [118].

Although proinflammatory cytokines may negatively affect the brain, IL-1β is still required for proper hippocampal memory function [119]. However, elevated IL-1β levels may dampen cognitive processing [120], as shown when the duration of impaired hippocampal-based cognition increased with elevation of hippocampal IL-1β levels [121]. Subsequently, an IL-1 receptor antagonist (IL-1RA) may block IL-1β activity, contributing to inflammation-induced cognitive deficits [122].

Circulating proinflammatory cytokines (due to HFD-induced systemic inflammation) can cross the mediobasal hypothalamus in the brain, activating cytokine receptors [123,124,125]. Cytokines and free fatty acids modulate the perpetuation of this inflammatory signal by producing local proinflammatory cytokines [125]. Additionally, excessive accumulation of free fatty acids may trigger central nervous system inflammation by activating a cascade of prostaglandins in centrally projecting neurons [126]. Therefore, systemic inflammation increases blood-brain barrier permeability, which allows immune cells and peripheral cytokines to enter the circulation [127]. A meta-analysis involving 5,717 participants and 1,311 cases suggested that increased inflammatory markers, such as C-reactive protein (HR = 1.45; 95% CI = 1.10, 1.91) and IL-6 expression (HR = 1.32; 95% CI = 1.06, 1.64) were associated with a modest increase in dementia risk [128].

## 6. Cognitive Impairment and Hypothalamic Inflammation

The hypothalamus is vital in the modulation of energy homeostasis by regulating energy expenditure and food intake [129]. The hypothalamus is responsible for a broad spectrum of physiological functions, such as reproduction, water balance, regulation, metabolism and feeding, and cardiovascular function. Furthermore, the hypothalamus is also involved in the memory aspects of cognition, learning, and attention [130]. For example, dysregulation of the hypothalamic pituitary adrenal (HPA) axis may impair the cognitive function [131]. Depressive patients have impaired executive function and memory recall, which is related to morning cortisol levels [132].

### Animal Study

Hypothalamic inflammation is an early acute response to high concentrations of free fatty acids [133]. Thaler et al. [66] reported that hypothalamic inflammation was observed within 1–3 days after HFD administration prior to body weight gain. Data further revealed that microgliosis, astrogliosis, and neuronal injury are initiated within the first week of HFD administration [66]. Glial cell activation is an essential step in HFD-induced hypothalamic inflammation through astrocyte and microglial reactivity [134]. Microglial activation and early markers of inflammation, such as TNF-α are often present in the ARC of the hypothalamus [64]. Microglia have been suggested as sensors of diet-induced hypothalamic inflammation because they express TLR4 to recognize long-chain fatty acids [135].

Astrocytes are the most predominant glial cell population within the central nervous system that responds to reactive astrogliosis [136]. Astrocytes produce cytokines and trigger inflammatory responses within the hypothalamus [137]. Astrocytes also control central nervous system infiltration and regulate oligodendrocyte and microglial activity [138]. A previous study suggested that central inflammation extends beyond the hypothalamus after feeding with a HFD, subsequently affecting areas related to cognition [111]. Moreover, an animal study demonstrated that administration of cultured primary microglia with sera derived from aged obese mice significantly enhanced oxidative stress and triggered microglia activation [139]. Upon activation, microglia undergo significant morphological changes. After one week administration of a HFD, microglial show reactive gliosis with significant proliferation [66], and subsequently may impact neuronal and synaptic plasticity [104]. Notably, the microglial-related inflammation in diabetic rat hippocampus can lead to the elevation of β-amyloid protein and tau pathology characteristic of Alzheimer’s disease [140]. Furthermore, oxidative stress is reported to be closely associated with cognitive impairment, brain proinflammatory cytokine production, and astrocyte activation following HFD feeding [48,141]. This finding implies that inflammation may influence neuronal function during HFD by promoting oxidative stress [104]. Data from another animal study further demonstrated that HFD triggers TLR4 and endoplasmic reticulum stress signaling pathways [142], activating the c-Jun N-terminal kinase (JNK) [64] and inhibitor of κB kinase β/nuclear factor-κB (IKKβ/NF-κB) [143,144] pathways in the hypothalamus [66].

Furthermore, increased inflammatory responses in the hypothalamus may produce leptin resistance, leading to defective food intake in dietary fat-induced obesity [64]. Data from an animal study revealed that HFD-induced alterations of hypothalamic metabolites and energy metabolism, suggesting an extension of the inflammation pathology to a localized metabolic imbalance produced by HFD [145].

Chronic stress can cause an elevation of hypothalamic proinflammatory cytokine expression, as well as recruitment of peripherally-derived monocytes into the brain such as anxiety-related brain regions for example amygdala [146,147]. This effect is associated with anxiety-related behavior and potentially to anxiety-associated mood disorders, with a stress-sensitized monocyte response that may lead to excessive anxiety in mice [147,148]. TLR4 is a major molecular target for SFAs in the hypothalamus that stimulates intracellular signaling pathways to induce an inflammatory response and contributes to obesity [133]. A previous study reported that HFD upregulates the hypothalamic inhibitor of nuclear factor kappa B kinase subunit beta (IKKB)/NF-κB, which in turn reduces hypothalamic leptin signal transduction [144]. Likewise, data from a genetic study further demonstrated that blocking hypothalamic NF-κB signaling reverses hypothalamic leptin resistance and promotes weight loss and reduced food intake in HFD-induced obesity [142]. Evidence indicates that obesity-derived neuroinflammation affects brain structures, such as the cortex and hippocampus [149]. Furthermore, high-fat consumption also significantly upregulates hypothalamic IL-6, NF-κB, and TLR4 expression in HFD-induced rats [67]. Collectively, these data suggest that stimulation of hypothalamic inflammation is sufficient to control food intake and is more likely involved in the mechanism underlying the pathogenesis of HFD-induced obesity.

## 7. Cognitive Impairment and Neurotrophic Factors

Brain-derived neurotrophic factor (BDNF) is abundantly expressed in the cerebral cortex, hippocampus, and hypothalamus. It plays a central role in the maintenance and growth of neurons [150]. BDNF causes alteration of neuronal plasticity via modulation of growth-associated protein 43 (GAP-43), cAMP response element-binding protein (CREB), and synapsin 1. BDNF also facilitates the release of neurotransmitters, axonal formation and growth, and maintenance of presynaptic structures by phosphorylating synapsin 1 [151].

### Animal Study

Most studies indicate that HFD decreases BDNF levels, and this phenomenon may increase the risk of memory deficits. Data from an animal study demonstrated that HFD decreased hippocampal BDNF protein and mRNA expression [152]. The study also reported downstream effectors, such as *CREB*, hippocampal synapsin 1, and *GAP-43* mRNA levels were diminished when BDNF level was reduced. Nevertheless, the mechanisms underlying reduced BDNF levels in HFD are not fully understood, but it is hypothesized that oxidative stress, augmented by ROS formation [153], contributes to these phenomena, and leading to cognitive dysfunction [154]. Most studies have addressed HFD-induced changes in BDNF level via activation of inflammatory cytokines [155]. Research suggests that chronic HFD exposure is required for reduced neurotrophic factors, such as GAP-43, synapsin 1, BDNF, and CREB [156]. Similar dietary intake was also found to induce brain inflammation and obesity-induced metabolic dysfunction [157].

## 8. Conclusions

Oxidative stress produces excessive ROS, primarily due to imbalances in oxidative to reducing species. It has been suggested that the overproduction of ROS is linked to a high level of inflammation and leads to impaired cognitive function. In fact, the brain is prone to oxidative stress due to its harnessing of chemically diverse reactive species to modulate heterogeneous signaling pathways. Excessive energy, particularly from HFD, impairs cognitive function. The rapid onset of HFD-induced hippocampal deficits may induce high levels of inflammation in the central nervous system. Although the adverse effect of HFD on cognitive function has been demonstrated in both in vitro and in vivo studies, there are still some controversies that need to be elucidated by long-term clinical trials with large cohorts of the general population. Therefore, future studies are needed to delineate the precise mechanism of action to better elucidate the role of HFD on cognition. Based on the evidence of this review, there are several limitations including (1) small sample size [58], (2) short duration of the study [58]; (3) different amount of fat [70]; and (4) lack of evidence from clinical studies showing changes in cognitive functions and fat [57]. It would be useful to have a dose-response relationship of fat in neurodegenerative diseases or cognitive functions compared in the same study, by taking into consideration of other aspects such as genetic, lifestyle, and social context.

## Figures and Tables

**Table 1:** The effects of a HFD on cognitive function in animal models.

Animal Strains [Number of Animals]	The Diet Used	Findings	References
Male 4-week-old Wistar rats (*n =* 10)	Hyperlipidic diet	↑ TNF-α, IL-1β, and IL-6 expression at 16 weeks. ↑ Phospho-[Thr^183^]-c-Jun N-terminal kinase (JNK) and NFκB. ↓ Insulin-induced tyrosine phosphorylation of insulin receptor (IR) and insulin receptor substrate (IRS-2). ↑ Hypothalamic expression of SOCS-3.	[64]
Middle-aged (12 months old) male C57BL/6 mice (*n =* 40 (8–10 mice per group))	The initial study [Western diet for 21 weeks] The following study [high-fat lard diet for 16 weeks]	↑ Increase glial fibrillary acidic protein (GFAP) expression. ↑ TNF-α, IL-6, MCP-1, and Iba-1 expression. ↓ Cortical BDNF levels.	[48]
Male C57BL/6J mice (3 weeks old) (*n =* 120)	HFD for 20 weeks	↓ The ratio of brain to body weight. The hepatocytes of HFD-fed mice were distended by large cytoplasmic lipid droplets. ↑ Immune densities and TNF-α. Showed activated microglia in the hippocampus. ↑ Hippocampal 4-hydroxynonenal (4-HNE) expression. ↓ p-IR, hippocampal phospho-AMP-activated protein kinase (p-AMPK), and phosphoacetyl-CoA carboxylase (p-ACC). ↑ Escape latencies and swimming distance during training trials.	[65]
Weight-matched male Long-Evans rats (300–350 g; Harlan) or male C57BL/6 mice (20–25 g) (*n =* 16)	HFD for 20 weeks	↑ Expression of proinflammatory genes by approximately 50% in both hypothalamus and liver. ↑ Hypothalamic expression of mRNA encoding myeloid cell-specific markers *Cd68* and *Emr1* (which encodes F4/80). ↑ Microglial number in the rat arcuate nucleus (ARC).	[66]
Six-week-old outbred male Sprague-Dawley rats (*n =* 55)	HFD	↑ Hypothalamic expression of genes encoding *TLR4* and *NF-κB* mRNA. ↑ Hypothalamic *TNF-α*, *IL-1β*, and *IL-6* mRNA expression.	[67]
Male C57BL/6J mice (three-week-old) (*n =* 48)	Western diet group	↑ Hypothalamus *IL-6*, *IDO*, *TNF-α*, and *IFN-γ* mRNA expression induced by LPS. ↑ Brain KYN/TRP ratio.	[68]
Wistar naïve male rats (3 weeks old (juvenile groups) or 12 weeks old (adult groups) (*n =* 143)	HFD	Long-term memory disturbance. Impaired long-term spatial reference memory in Morris water maze. Showed a higher latency to reach the platform. Spatial reversal learning enhanced interleukin-1β (*IL-1β*) mRNA level in the hippocampus. ↑ *TNF-α* and *IL-6* mRNA expression.	[69]
C57BL/6J male mice (5-week-old, 15–20 g) (*n =* 34)	HFD	Animals treated with HFD for 48 h presented a partial inhibition of long-term potentiation (LTP). The maintenance of LTP was impaired by HFD treatment.	[50]
Male C57BL/6 mice (8-week-old) (*n =* 10)	HFD for 12 weeks	HFD did not alter the swimming speed, performance of mice in Morris water maze during learning phase or memory retrieval phase. ↓ Total travel distance. Induced anhedonia and despair in mice. ↑ Immobility in forced swimming test. ↑ GFAP in the whole hippocampi. ↓ The lengths of total process and the numbers of branch point of the GFAP^+^ astrocytes in the hippocampal CA1 and CA3 regions. ↓ Glutamate-aspartate transporter (GLAST), glutamate transporter-1 (GLT-1), and connexin 43 (Cx-43) in the hippocampi.	[70]
Male C57BL/6N mice (aged 6–8 weeks) (*n =* 297)	HFD	↑ Local inflammation such as CD53, leptin, interleukin 1 receptor antagonist, cathepsin S, integrin beta 2, chemokine (C-C motif) ligand 2, interleukin 7 receptor, and chemokine (C-C motif) ligand 8.	[71]
Male (C57Bl/6J mice) 12 week old (*n =* 112)	HFD for one day	Induce episodic memory, contextual associative, and spatial memory	[51]
Male (Orexin/ataxin-3 (O/A3) mice (7–8 month of age) (*n =* 36)	HFD	Learning impairment was evident in both 2 and 4 weeks. ↑ Microglial activation marker *Iba-1*. ↑ CX3 chemokine receptor 1 (*CX3CR1*), *TNF-α*, and mitochondria-associated enzyme immune responsive gene-1 (*Irg1*).	[52]

**Table 2:** The effects of a HFD on cognitive function in human studies.

Subjects [Sample Size]	Study Design	The Diet Used	Findings	Limitations	References
Elderly (*n =* 5395)	At baseline (1990 to 1993), the subjects had normal cognition, were noninstitutionalized, and underwent a complete dietary assessment by a semiquantitative food-frequency questionnaire (FFQ). The cohort was continuously monitored for incident dementia [(1993–1994) and (1997–1999)].	HFD (Total, saturated, trans fat, and cholesterol)	Not associated with increased risk of dementia or its subtypes.	The semiquantitative FFQ may not have enough precision to measure the nutrient intake.	[72]
Elderly individuals free of dementia (*n =* 980)	The subjects were followed for a mean of 4 years. Daily consumption of protein, fats, carbohydrates, and calories was recalled using a semiquantitative FFQ administered between the baseline and first follow-up visits.	HFD	A high intake of SFA was not associated with the increased risk of Alzheimer’s disease.	The semiquantitative FFQ may not have enough precision to measure the nutrient intake. Underreport calorie intake.	[73]
Nondemented elderly subjects (65–84 years) (*n =* 704)	Participants were followed-up for a median period of 2.6 years. Dietary intakes were assessed at baseline with a 77-item semiquantitative FFQ.	High PUFA	Protect against the development of mild cognitive impairment.	Small sample size. The length of the follow-up time was fairly short. The nonparticipation rate was high. The attrition rate of this longitudinal study is quite high.	[61]
Healthy male adults (aged 22 ± 1 year) (*n =* 16)	Subjects were randomly assigned to receive either a high-fat, low-carbohydrate diet or a standard diet for 5 days and then crossed over to the alternate diet after a 2-week washout period. During the diets, cognitive function was measured.	High-fat, low-carbohydrate diet for 5 days	Impaired attention, speed, and mood.	Small sample size.	[57]
Sedentary men with fewer than 2 h/week physical activity (*n =* 20)	The participants were assessed when consuming a standardized, nutritionally balanced diet (control) and after 7 days of consuming a diet comprising 74% kcal from fat.	HFD	Increased simple reaction times and decreased power of attention.	Small sample size.	[58]
Elderly (*n =* 6183)	Serial cognitive testing was conducted over 4 years, began 5 years post-dietary assessment using 131-item semiquantitative FFQ.	High SFA	↓ Global cognitive and verbal memory trajectories.	Repeated diet assessments were not available. Reverse causation is possible. The participants were primarily Caucasian women.	[41]
Elderly (median age, 79.5 years) (*n =* 937)	Participants were followed over a median of 3.7 years of follow-up. At baseline and every 15 months, participants were evaluated using the Clinical Dementia Rating Scale, a neurological evaluation, and neuropsychological testing for a diagnosis of dementia, normal cognition, or mild cognitive impairment. The participants were given a 128-item FFQ at baseline.	HFD	↓ The risk of mild cognitive impairment or dementia.	Recall bias in reporting of dietary nutrients. The participants were primarily northern European ancestry. Non-participation bias. FFQ may not have enough precision to measure nutrient intake.	[63]
